# Atomic and electronic structure of twin growth defects in magnetite

**DOI:** 10.1038/srep20943

**Published:** 2016-02-15

**Authors:** Daniel Gilks, Zlatko Nedelkoski, Leonardo Lari, Balati Kuerbanjiang, Kosuke Matsuzaki, Tomofumi Susaki, Demie Kepaptsoglou, Quentin Ramasse, Richard Evans, Keith McKenna, Vlado K. Lazarov

**Affiliations:** 1 Department of Physics, University of York, Heslington, York, YO10 5DD, UK; 2 Secure Materials Centre, Materials and Structures Laboratory, Tokyo Institute for Technology, 4259 Nagatsuta, Midori-ku, Yokohama-city, Kanagawa, 226-8503, Japan; 3 SuperSTEM, STFC Daresbury Laboratories, Keckwick Lane, Warrington, WA4 4AD, UK

## Abstract

We report the existence of a stable twin defect in Fe_3_O_4_ thin films. By using aberration corrected scanning transmission electron microscopy and spectroscopy the atomic structure of the twin boundary has been determined. The boundary is confined to the (111) growth plane and it is non-stoichiometric due to a missing Fe octahedral plane. By first principles calculations we show that the local atomic structural configuration of the twin boundary does not change the nature of the superexchange interactions between the two Fe sublattices across the twin grain boundary. Besides decreasing the half-metallic band gap at the boundary the altered atomic stacking at the boundary does not change the overall ferromagnetic (FM) coupling between the grains.

Atomistic control of defects and phase segregation in technological materials is an essential challenge for realising predicted functionality in materials science^1^, particularly in structural oxides including perovskites, spinels and manganites^2^. Extended defects in oxides influence many important properties for technological applications including magnetic, ferroelectric, and electron transport behaviour. The presence of such defects is well-known but it is experimentally challenging to resolve their atomic-scale structure, which is a crucial step in understanding the role of a particular defect on the functional properties of the studied material.

In magnetic oxides any disturbance to the crystal structure is liable to significantly alter properties such as conductivity and magnetic ordering. The overall properties in those materials are strongly dependent on the local atomic co-ordination and structure due to the local nature of electron hopping mechanisms and super exchange interactions.

A typical example of a structural defect in Fe_3_O_4_ are the antiphase boundaries (APB). Such a defect breaks the translational symmetry of the crystal lattice which results in altered bond lengths and angles. These structural changes result in the presence on non-bulk superexchange interaction in the boundary vicinity which consequently drastically modifies the magnetic and electronic properties of thin film magnetite, hence they have been extensively studied due to relevance for device applications^3^,^4^,^5^,^6^,^7^,^8^.

In this work we focus on a different type of structural boundary, the twin boundary, which we observed in thin films of Fe_3_O_4_. Twin defects are common in face centred cubic (*fcc*) materials and are observed across a wide range of natural^9^,^10^ and synthetic specimens^11^,^12^. Such defects are essential to the mechanical behaviour of materials e.g. shape memory alloys^13^ and they mediate stress and strain in both functional and mechanical materials. However their effect on magnetic properties has rarely been studied. This is mainly due to the lack of atomic-scale structural models of twin defects in spinels. Recent progress in the growth of high quality thin films of magnetite with bulk like properties^14^ and atomic level transmission electron microscopy characterisation methods have provided opportunities for detailed atomic level studies that has enabled correlation between the defects structure and film functional properties^15^.

Here we have determined the atomic structure of a twin defect in Fe_3_O_4_ thin films. In particular we studied the atomic arrangement at the (111) twin boundary and its effect on the magnetic and electronic properties of Fe_3_O_4_. Fe_3_O_4_ is a prototype material for spinel metal oxide structure systems; therefore the findings in this work are relevant for number of spinel based oxides.

Before we present the main findings we shortly review the basic properties of Fe_3_O_4_. Fe_3_O_4_ forms a cubic inverse-spinel structure, with lattice constant a_0_ = 0.834 nm and space group Fd-3m^16^. The Fe sites are divided into one tetrahedral ‘Fe_A_’ and two octahedral ‘Fe_B_’ sites per formula unit. The net magnetic moment of Fe_3_O_4_ arises due to the opposing magnetisation of the two Fe_B_ with respect to the Fe_A_ site which leaves an uncompensated magnetic moment of 4 μ_B_ per formula unit. The magnetic order of Fe_3_O_4_ is driven by superexchange interactions across Fe-O-Fe bonds. The high Curie temperature of ~858K together with predicted 100% spin polarisation at the Fermi level from electronic structure calculations^17^,^18^ makes magnetite very attractive for various spin-electronic devices.

The thin films of magnetite studied in this work were prepared by pulsed laser deposition (PLD) on yttria-stabilised ZrO2 (YSZ) substrate. The film structure was optimized by CO_2_/CO post annealing^14^; details are presented in experimental methods and supplementary data, Figures S1–S3. Figure 1a shows a cross-section of the grown film that includes the grain boundary. This medium angle annular dark field (MAADF) scanning transmission electron microscopy (STEM) image outlines a (111) oriented defect in a plane normal to the growth direction of Fe_3_O_4_(111)/YSZ(111) heterostructure. The defect can be clearly identified by the higher contrast (due to strain-induced contrast in MAADF imaging^19^) line, running horizontally through the Fe_3_O_4_ layer (highlighted with yellow dashes). High angle annular dark field (HAADF) image from the same region is shown in Figure S4. In all cases of twinning along the (111) growth front we observe an extended grain boundaries often propagating for 200–300 nm on a single (111) atomic plane. The overall morphology of the twin grain shown in Fig. 1a is typical. The selected area diffraction (SAD) pattern shown in Fig. 1b demonstrates the single crystal spinel structure of the grown Fe_3_O_4_ film. The strain emerging due to the large lattice mismatch between the film and substrate (the lattice constant of Fe_3_O_4_ is 19% smaller than a double lattice constant of YSZ) is released through the formation of misfit dislocations; hence the film is fully relaxed and epitaxially related to the YSZ(111) substrate with a standard cube to cube epitaxy. The rhombohedral diffraction pattern, characteristic for the cubic structures along the 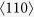 type of directions, shows the substrate (white rhombus and indices), untwinned (red rhombus and indices) and the twinned regions of the film (blue rhombus and indices). The epitaxial relationship between the film and substrate is defined by the following relations: Fe_3_O_4_(111)||YSZ(111) and Fe_3_O_4_(1–10)||YSZ(1–10). The twin nature of the defect presented in Fig. 1a can be readily recognized by the mirrored diffraction pattern of the Fe_3_O_4_ along the growth [111] direction (labelled in blue).

Next we perform atomically resolved imaging in order to determine the atomic environment in the vicinity of the twin boundary. Figure 2a shows a HAADF STEM image of the Fe_3_O_4_ upper and lower grain around the (111) defect. The overlaid structural model outlines the Fe atomic columns above and below the twin defect, with tetrahedral Fe_A_ sites in yellow and octahedral Fe_B_ sites in red. The octahedral sites are further divided into double (high intensity) and single (low intensity) occupancy sites. The distinctive spinel stacking motif consisting of …/4O/Fe_A_Fe_B_ Fe_A_/4O/3Fe_B_/4O/… along the [111] growth direction is clearly seen in upper and lower grain. By following the intensities of Fe atomic columns the reversal in stacking at the boundary is easily identified. We use double yellow dashed line to separate the fully stoichiometric grains above and below the defect plane. We note that the upper and lower grains are geometrically related by mirror plus shift vector of *a*/6[1,–2,1], where *a* is the lattice constant of magnetite. Once the region of the twin boundary is uniquely determined we focus on the detailed atomic and chemical arrangement at the boundary (region between the two-dashed yellow-lines.)

Figure 2b shows atomically resolved HAADF image from the twin boundary region. When this region is compared to the bulk counterpart (Fe_A_-Fe_B_-Fe_A_), i.e. atomic planes away from the defect boundary, it is observed that at the twin boundary only two atomic columns are occupied, both tetrahedral Fe_A_ (as determined by the structural position). This gives the boundary a chemical layering of …Fe_A_-Fe_B_-Fe_A_/4O/3Fe_B_/4O/**Fe**_**A**_**-Fe**_**A**_/4O/3Fe_B_/4O/Fe_A_-Fe_B_-Fe_A_… with a typical reversal in the *fcc* like stacking across the twin …*ABC-BAC*… observed across this defect. The missing Fe_B_ plane (columns positions indicated by the open red circles in Fig. 2b) makes the twin boundary non-stoichiometric.

The impact of the missing Fe plane at the twin boundary was further investigated by electron energy loss spectroscopy (EELS) measurements, an example of which is shown in Fig. 3 and Figure S5. 2D spectrum images were acquired by rastering the electron probe serially across a defined region across the twin boundary (Fig. 3a) and collecting an EEL spectrum at each point. Chemical maps of O and Fe (Fig. 3b) were created by integrating at each point of these spectrum images the spectrum intensity over an ~40 eV window above the O *K* and Fe *L*_*2,*3_ edge onsets, respectively, after background subtraction using a power law model. While the O map (Fig. 3b) shows a rather uniform intensity across the twin boundary area, the Fe map (Fig. 3b) shows a drop of intensity along the twin boundary area, indicating depletion in the Fe content. This relative drop of the Fe signal can be readily seen by plotting intensities of the O and Fe signals (integrated over area marked by the dashed line in Fig. 3b), against the integrated HAADF signal acquired simultaneously with the EELS measurements, as shown in Fig. 3c. Note that the overall intensity gradient of the HAADF and EELS signal intensity profiles (the increase from left to right in Fig. 3c) is due to specimen thickness variation from the film surface (left side in Fig. 3a,b) towards the substrate (right side in Fig. 3a,b). The integrated Fe *L*_*2,3*_ and O *K* EELS signal profiles follow the increasing trend of the HAADF-STEM image intensity profile, as expected from the increasing specimen thickness. The HAADF intensity profile however shows an abrupt drop in intensity at the twin defect region. Furthermore, at the defect boundary the intensity of the Fe signal (red curve in Fig. 3c) drops while that of O (blue curve in Fig. 3c) remains overall unaffected.

The depletion in Fe at the twin defect is observed by subtle variations in the near edge structure (ELNES) of both the O *K* (Fig. 3d) and the Fe *L*_2,3_ edges (Fig. 3e). The O *K* ELNES of Fe oxides displays four distinct peaks (labelled as (a–d) in Fig. 3d); the relative intensity of some of these features, namely peaks (a) and (c) is shown to be dependent on the relative Fe and O ratio and structure of the Fe oxide^20^. More specifically, the O *K* spectrum integrated from an area along the twin boundary (designated as area 2 in Fig. 3b,c) shows a slight increase in intensity of the peak ‘a’ and smearing of the peak ‘c’ (Fig. 3c)^20^ compared to the integrated O *K* spectra from either side of the twin boundary (designated as areas 1 and 3 in Fig. 3b,c and spectra O1 and O3 in Fig. 3d). The spectra have been normalised to the (b) peak for reading clarity. These fine structure changes indicate that the local chemistry at the twin boundary is shifted from the Fe_3_O_4_ bonding environment to a more α-Fe_2_O_3_ like bonding environment^20^, which agrees with the observation of Fe depletion at the twin boundary. This effect is further corroborated by a change in the relative intensity of the Fe of the *L*_*3*_ and *L*_*2*_ peaks at the twin boundary region; the spectra shown in Fig. 3e are integrated over the same areas as the O *K* spectra in Fig. 3d, and normalised for reading clarity to the maximum of the *L*_*3*_ peak. The relative drop of the *L*_2_ intensity at the twin boundary (spectrum Fe2 in Fig. 3e) compared to either side of the twin boundary (spectra Fe1 and Fe3 in Fig. 3e) are characteristic of small increase in the Fe valence^20^ in agreement with a change from Fe_3_O_4_ to a Fe_2_O_3_-like bonding environment at the boundary. It should be noted that while these changes in the near edge fine structures of the O *K* and Fe *L*_*2,3*_ edges are arguably small, they are systematically observed across several datasets (Figure S5). Even though the EELS at the twin boundary is subtle and requires a careful analysis of the spectra, all the implied conclusions are fully consistent with the atomic resolution HAADF imaging presented above, e.g. Fig. 2b.

It is of interest to consider what effect the changes in local chemistry and atomic structure at the twin boundary will have on the magnetic/electronic properties of the Fe_3_O_4_ films. It is well documented that disruptions in the superexchange interactions due to antiphase domain boundaries can drastically affect the film properties^3^,^7^,^21^. For this purpose, based on the experimental data we build atomic models for density functional theory (DFT) calculations in order to determine both the atomic structural refinement of the twin defect and its electronic structure.

One of the constraints in performing the DFT calculations on the twin boundary is to account for non-stoichiometry associated with the missing Fe_B_ layer at the boundary. The missing ionic Fe_B_^2.5+^ layer means the interface is effectively negatively charged. This charge can be compensated by various means. One possibility is electronic compensation through the localisation of electron holes (which may reduce Fe or O ions near the interface). Another option is through atomic defects, either intrinsic (such as oxygen vacancies) or extrinsic impurities which are not observed by the EELS chemical analysis. Since in the DFT supercell one Fe_B_ atom is missing from each interface with a formal charge of +2.5 the net charge required to compensate the two twin defects in the supercell is +5. Here we consider electronic compensation by introducing electron holes at the boundary.

Figure 4a shows the relaxed atomic model of the twin in the electronically hole-compensated structure. In this model the ferromagnetic alignment of the adjacent domains is preferred. The energy required to align the magnetisation of the two structural domains antiparallel i.e antiferromagnetic coupling is 133 mJ/m^2^. The spin polarised density of states (SDOS) are shown in Fig. 4d and compared to the bulk magnetite SDOS. There is a clear shift of majority band to higher energy (relative to the Fermi level) which reduces the size of the half-metallic band gap and introduces a small amount of interfacial states. However a switch of the spin polarization across the boundary as is the case in [110] anti-phase boundary is not present^15^. This result indicates that even though the overall magnetic properties of the film are not affected by the twin boundary, a loss of the half-metallicity at the boundary is very likely at elevated temperatures due to reduced value of the band gap. In addition to the electron-hole compensated mechanism for twin boundaries we have considered a H (proton) stabilised twin which yields very similar results to the hole-compensated model, both in terms of structure, electronic properties and magnetic alignment.

The structurally converged model from DFT was used to calculate HAADF intensity map at the interface. The simulated HAADF image (Fig. 4b) shows a good correspondence with the experimental HAADF image (Fig. 4c) from the same interface area. The missing Fe_B_ plane at the boundary (as illustrated in Fig. 2b) is clearly seen in both images (Fig. 4b,c).

The DFT prediction that the twin grains are ferromagnetically coupled follows from the local atomic bond arrangements at the twin boundary with missing Fe_B_ plane. Compared to APB defects in Fe_3_O_4_ in which the bulk-like 90° coupling across Fe_B_-O-Fe_B_ bonds shifts to 180° leading to strong anti-ferromagnetic (AFM) coupling, the twin structure observed here does not generate any non-bulk high-angle bonding. Across the twin defect the dominant 125.3° Fe_A_-O-Fe_B_ bulk superexchange^7^,^22^,^23^ interaction is not modified implying that the overall ferrimagnetic order of the two Fe_A_ and Fe_B_ sublattices continues through the twin defect undisturbed. However, due to the missing Fe_B_ plane, the number of Fe_A_-O-Fe_B_ superexchange interactions at the boundary is reduced. This results in weaker coupling between the octahedral and tetrahedral sublattices which could lead to domain wall pinning at this type of boundary.

In addition to non-stoichiometric twin boundaries we have also observed the presence of stoichiometric twin boundaries. The appearance of both stoichiometric and nonstoichiometric boundaries is most likely related to kinetic effects, however the exact mechanism of their formation is not known. Finally, we note that besides twin boundaries the film also contains many APBs, which are the main cause of the anomalous properties of the films.

In this work we have shown that twin defects are present in thin film Fe_3_O_4_(111)/YSZ(111). The twin boundary is on (111) growth planes and it is formed by the breaking of symmetry of a Fe_A_-Fe_B_-Fe_A_ layer. Aberration-corrected HAADF imaging shows that the boundary is non-stoichiometric with a missing Fe_B_ plane. Electron energy loss spectroscopy shows changes in Fe and O core edges as well as depletion of the Fe at the boundary, which compliments the HAADF results. The DFT calculation of this non-stoichiometric boundary structure was modelled by introducing electron holes as a charge-compensation mechanism to realise the ionic nature of Fe_3_O_4_. The electronic calculations show that majority band gap is significantly reduced with a presence of the interface states. Atomic bond counting from the DFT-optimised geometrical coordinates shows no presence of high-angle Fe-O-Fe bonds hence the absence of AFM superexchange interactions at this boundary in comparison to APBs in ferrite spinels. The FM coupling between the twin grains was also confirmed by the DFT calculation which found that AFM coupling is less energetically favourable compared to FM coupling. This work clearly shows that the (111) oriented non-stoichiometric boundaries are energetically stable and their effect is more subtle in comparison to the antiphase domain boundaries.

## Experimental Methods

Fe_3_O_4_ films of 100 nm thickness have been grown on (111) oriented yttria-stabilised zirconia (YSZ) by pulsed laser deposition (PLD) with a KrF excimer laser incident on a stoichiometric sintered Fe_3_O_4_ target. During deposition the substrate was held at 300 °C in an oxygen partial pressure of 2 × 10^−4 ^Pa. The post-annealing in a CO/CO_2_ atmosphere removes all secondary phase usually seen due to polarity of the Fe_3_O_4_ (111) films^24^,^25^, and also significantly improve the stoichiometry and atomic ordering in the grown films^14^.

Cross sectional TEM specimens have been prepared by conventional means including mechanical polishing and Ar ion-thinning to electron transparency^26^. Serial acquisition of stack of images followed by rigid registration *via* the SDSD Digital Micrograph plug-in were used to provide significantly improved signal-to-noise ratio without compromising drift or risking specimen damage^27^.

Transmission electron microscopy observations and electron diffraction have been performed using a JEOL 2011 operated at 200 kV. HAADF-STEM imaging was performed using a JEOL JEM 2200-FS operated at 200 kV. Further HAADF-STEM imaging and EELS were performed using the Nion UltraSTEM 100 which is equipped with a Gatan Enfina spectrometer. The microscope was operated at 100 kV acceleration voltage and the probe-forming optics were configured to form a ~0.9 Å probe (full width at half-maximum) with a convergence angle of 30 mrad and a probe current of ~120 pA. The semi-angular range of the HAADF and MAADF detectors were 86–190 and 40–86 mrad, respectively. The native energy spread of the electron probe was 0.3 eV and the collection semi-angle for the electron energy loss spectroscopy measurements 36 mrad. Spectra were collected at a 0.3 eV/channel dispersion, yielding an effective energy resolution of 0.9 eV.

In order to minimize the effect of elastic scattering in the EELS signal, the EELS spectra were acquired at a relatively large collection angle (36 mrad). The relative thickness of the sample in the areas of interest did not exceed 0.5∙t/λ (where t the thickness and λ the electron free mean path for 100kV electron beam), as determined by using low loss EELS spectrum images of identical dimension, acquired subsequently in the exact same areas as the core-loss data. As a result, the EELS spectra were not further treated for multiple scattering effects.

In order to assess the effect of multiple scattering in the EELS signal, low loss EELS spectrum images were systematically acquired at the same areas as the core loss signal. Post mortem images were acquired immediately after each EELS acquisition to assess possible beam damage effects. The data presented in the manuscript are as acquired, with no post processing, apart from background subtraction. In order to minimise the electron beam damage electron dose distributed EELS SMART acquisition technique was employed^28^, yielding very similar results (see Figure S5).

## Theoretical Methods

We predict the stable structures of extended defects in Fe_3_O_4_ using a two-stage modelling approach as employed in previous studies of defects in MgO, TiO_2_ and HfO_2_ and Fe_3_O_4_^29^,^30^. Potential defect structures are first screened using a classical pair potential to identify the most stable candidates before final refinement at the density functional theory level. Two equivalent (111) twin defects are introduced into a periodic supercell and the total energy is optimised with respect to the positions of all ions, the length of the supercell in the direction perpendicular to the (111) planes and the rigid translations of one domain with respect to the other. Spin-polarised density functional theory calculations are performed on the most stable candidate structures using the projector augmented wave method as implemented in the VASP code. The PBE functional for exchange-correlation is employed and wavefunctions are expanded in a plane wave basis with energies up to 400 eV^31^,^32^,^33^. For the bulk Fe_3_O_4_ primitive unit cell a 7×7 × 7 k-point grid is employed and the bulk lattice constant is predicted to be 8.40 Å. Similar k-point densities are employed for the supercell calculations and the cell dimensions in the (111) are plane fixed in proportion to the appropriate bulk lattice constant. The separation between periodically replicated (111) twin defects in these calculations was 33.9 Å which was sufficient to ensure convergence to bulk-like properties in the centre of the grains. These approaches proved reliable for predicting the structure and stability of antiphase defects in Fe_3_O_4_ in a previous study^15^.

HAADF STEM image simulations of the twin boundary have been performed using the relaxed structural coordinates provided by DFT calculations, using the multi-slice method implemented in the QSTEM image simulation suite^34^,^35^. Simulations have been performed using 30 thermal diffuse scattering iterations and experimentally determined electron beam parameters relating to the JEOL 2200-FS of, acceleration voltage = 200 kV, chromatic aberration C_C_ = 1.6 mm, spherical aberration C_s_ = 0.0011 mm, fifth-order spherical aberration C_5_ = 1.756 mm, convergence semi-angle α = 24 mrad and HAADF-STEM detector acceptance semi-angle 85–170 mrad^15^.

All data created during this research are available by request from the University of York Research database.

## Additional Information

**How to cite this article**: Gilks, D. *et al.* Atomic and electronic structure of twin growth defects in magnetite. *Sci. Rep.* **6**, 20943; doi: 10.1038/srep20943 (2016).

## Supplementary Material

Supplementary Information

## Figures and Tables

**Figure 1:**
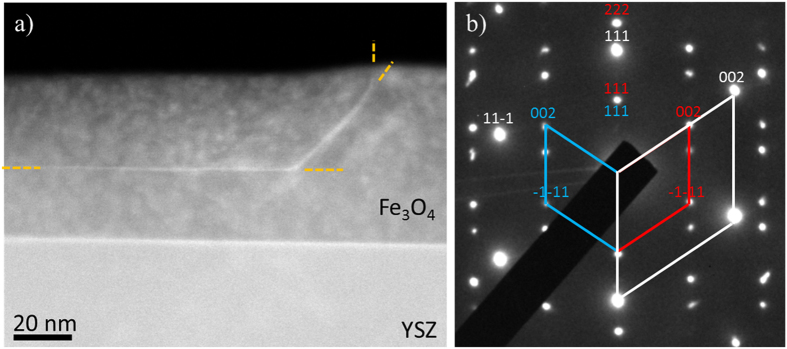
(**a**) Low magnification MAADF-STEM image of the Fe_3_O_4_/YSZ(111) interface with a (111) oriented twin structure propagating horizontally through the deposited layer, highlighted with yellow dashes for clarity. In these images, the brighter contrast of the defect originates from local strain. (**b**) SAD pattern taken from a twinned region showing the characteristic rhombohedral pattern associated with the [1–10] zone axis of both YSZ and Fe_3_O_4_. The brightest reflections highlighted with the white rhombus and white diffraction indices are from the substrate. The two smaller rhombohedral constructions in red and blue are from the two grains in the Fe_3_O_4_ film and indicate to twinned nature of the boundary.

**Figure 2:**
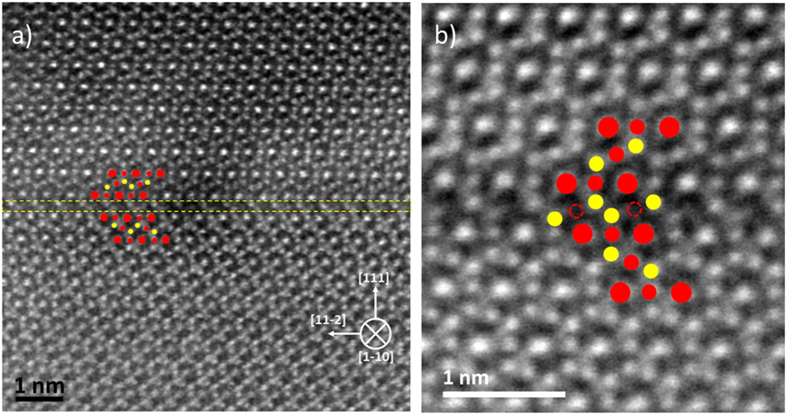
(**A**) HAADF-STEM image showing the twin defect region outlined by the two dashed yellow lines. The bulk like structure of the upper and lower grain is outlined with the overlaid dots; where large (small) red dots represents octahedral double (single) occupied Fe atomic columns and yellow dots represent single occupied tetrahedral site. (**B**) Atomic resolution HAADF-STEM image of the (111) twin defect boundary obtained by rigid registration of 26 consecutive rapidly acquired images. The columns positions of the missing Fe octahedral plane are outlined by the red-dashed circles. The overlaid structural model follows the colour code from Fig. 2.

**Figure 3:**
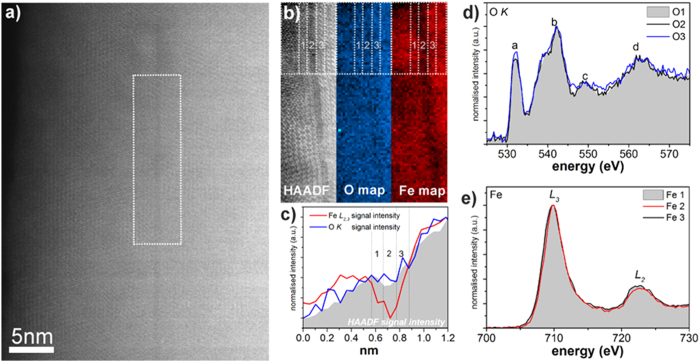
(**a**) HAADF STEM survey image of the Fe_3_O_4_ twin boundary; the area selected for 2D EELS mapping is marked with white rectangle. (**b**) HAADF STEM signal acquired simultaneously with the EELS signal and 2D intensity maps of the O *K* and Fe *L*_*2,3*_ EELS signal, (**c**) Intensity profiles of the Fe *L*_2,3_ (red) and O *K* EELS (blue) integrated over the area marked by the rectangle in (**b**), plotted along with the HAADF image intensity profile (grey). The drop in the Fe signal along with the small drop of the HAADF image intensity are indicative of Fe depletion at the twin boundary. Normalised (**d**) O *K* and (**e**) Fe *L*_2,3_ EELS spectra acquired at and adjacent to the twin boundary (marked as (2) and (1,3) respectively). The increase of peak ‘a’ and loss smearing of peak ‘c’ in O *K ELNES* edge together with the small increase of the I_L3_/l_L2_ ratio at the boundary indicate to a slight increase of the Fe oxidation state^20^ (consistent with the drop of the Fe signal).

**Figure 4:**
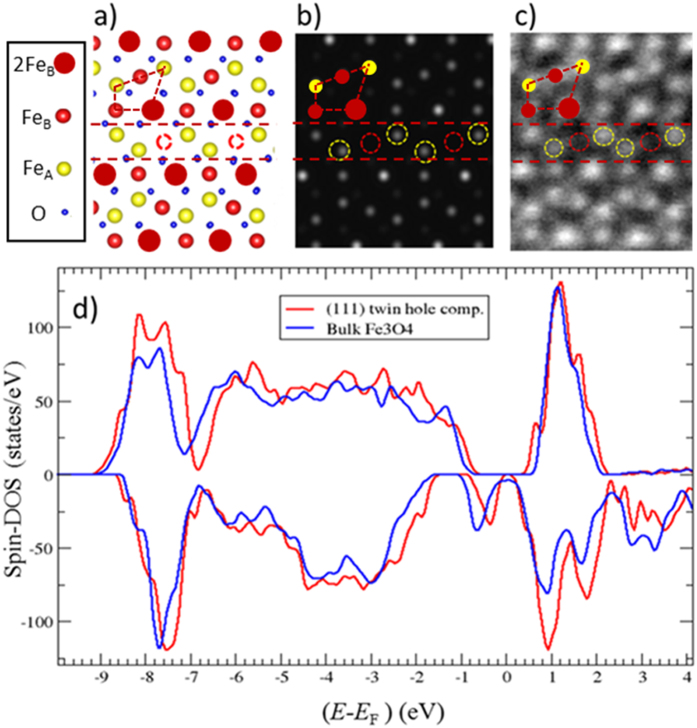
(**a**) Structural model of the non-stoichiometric (111) twin defect stabilized with electron holes. The position of the missing Fe_B_ plane from the defect region is denoted by open red circles. The colour coding (also presented in the legend) is the following: yellow- Fe_A_; red (big) – double occupied Fe_B_; red (small) – single occupied Fe_B_; blue – oxygen. b) HAADF-STEM image simulation produced using the structure in (**a**). (**c**) Experimental HAADF STEM image covering the same film region as in (**a,b**). The two dashed red lines outline the defect region. Open yellow circles outline the FeA atomic columns positions (within the defect region), while open red circles the positions of the missing Fe_B_ plane. The region outlined with the dashed polygon in (**a**) is overlaid in (**b**) and (**c**) to demonstrate the matching between the HAADF images and the structural model presented in (**a**). Note that due to the Z – contrast of the HAADF STEM imaging oxygen is almost invisible in (**b,c**). (**d**) Spin-resolved density of states of the hole compensated (111) twin plotted along with SDOS from bulk magnetite.
