# Supplementary material for: Atomic and electronic structure of twin growth defects in magnetite
Source: Sci Rep. 2016 Feb 15;6:20943. doi: 10.1038/srep20943 (PMC4753457; doi:10.1038/srep20943)
Supplement: Supplementary Information [file srep20943-s1.pdf]

Atomic and electronic structure of twin growth defects in magnetite

*Daniel Gilks[1], Zlatko Nedelkoski[1], Leonardo Lari[1], Balati Kuerbanjiang[1], Kosuke Matsuzaki[2], Tomofumi Susaki[2], Demie Kepaptsoglou[3], Quentin Ramasse[3], Richard Evans[1], Keith McKenna[1], and Vlado K. Lazarov[1]**

1. Department of Physics, University of York, Heslington, York, YO10 5DD, UK.
2. Secure Materials Centre, Materials and Structures Laboratory, Tokyo Institute for Technology, 4259 Nagatsuta, Midori-ku, Yokohama-city, Kanagawa, 226-8503, Japan.
3. SuperSTEM, STFC Daresbury Laboratories, Keckwick Lane, Warrington, WA4 4AD, UK.
*E-mail: vlado.lazarov@york.ac.uk

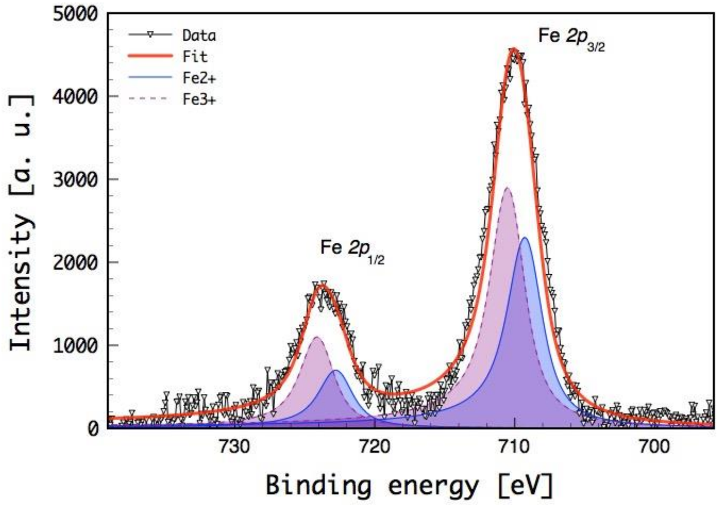

Supplementary Figure S1. XPS results from Fe_3O_4/YSZ(111). The peak positions of Fe^{2+} ($2p_{3/2}$) at 709.5 ± 0.2 eV and Fe^{3+} ($2p_{3/2}$) at 710.7 ± 0.2 eV are determined after fitting the data. These results agree well with the literature values of magnetite within the error bar [NIST XPS database: http://srdata.nist.gov/xps/main_search_menu.aspx].

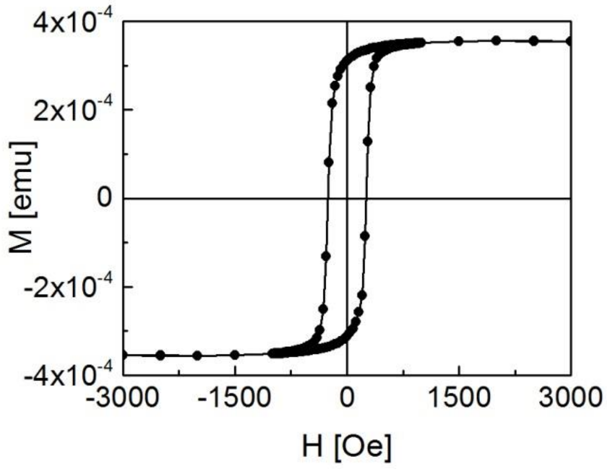

Supplementary Figure S2. Hysteresis of the annealed Fe_3O_4(111)/YSZ(111) sample at room temperature.

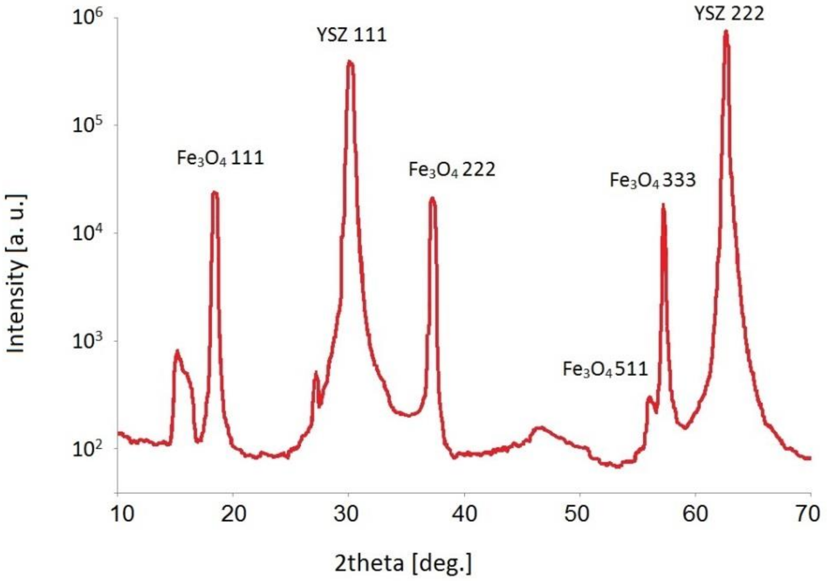

Supplementary Figure S3. XRD from annealed Fe_3O_4(111)/YSZ(111) sample.

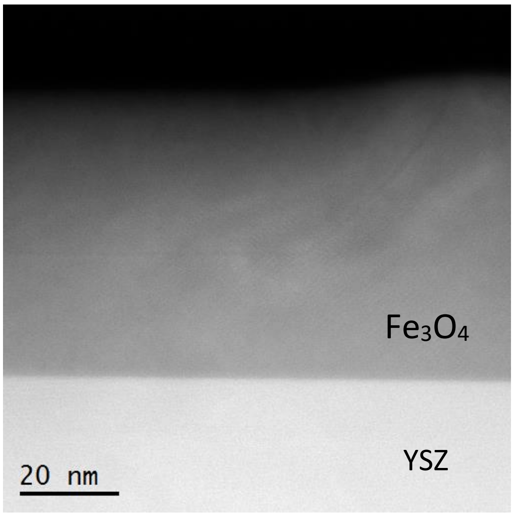

Supplementary Figure S4. HAADF image from the twin boundary region presented in Figure 1a. Note that the MAADF image (Figure 1a) and the HAADF image are recorded simultaneously.

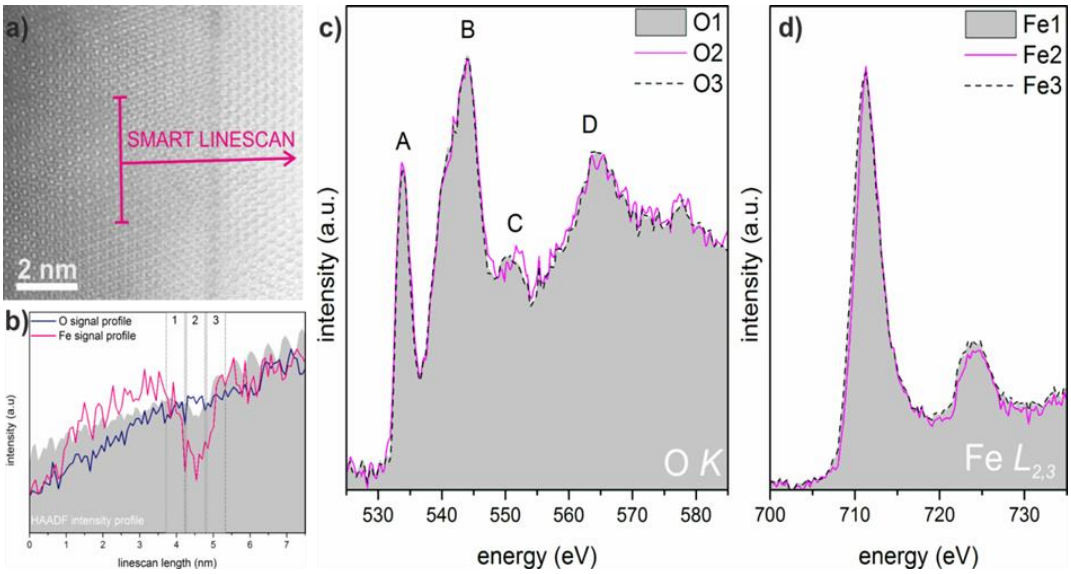

Supplementary Figure S5. (a) HAADF STEM image and schematic of the SMART EELS scan [20] across the twin boundary: EEL spectra are acquired serially along the direction marked with the arrow while the beam is distributed along the perpendicular direction; (b) plots of the Fe $L_{2,3}$ (pink) and O *K* EELS (blue) signals along the linescan, plotted against the HAADF image intensity profile (grey). The drop in the Fe signal along with the small drop of the HAADF image intensity are indicative of Fe depletion at the twin boundary. (c) O *K* and (d) Fe $L_{2,3}$ spectra acquired at (pink) and adjacent to (grey/blue) the twin boundary (marked with dashed lines in panel (b)). The small shift of peak 'C' in the O *K* edge to higher energies and a small increase of the I_{L3}/I_{L2} ratio at the boundary indicate an increase of the Fe oxidation state [21] (consistent with the drop of the Fe signal).
